# *IRE1*-Independent Gain Control of the Unfolded Protein Response

**DOI:** 10.1371/journal.pbio.0020235

**Published:** 2004-08-17

**Authors:** Jess H Leber, Sebastián Bernales, Peter Walter

**Affiliations:** **1**Department of Biochemistry and Biophysics, University of CaliforniaSan Francisco, California, United States of America; **2**Howard Hughes Medical Institute, Chevy ChaseMarylandUnited States of America

## Abstract

Nonconventional splicing of the gene encoding the Hac1p transcription activator regulates the unfolded protein response (UPR) in Saccharomyces cerevisiae. This simple on/off switch contrasts with a more complex circuitry in higher eukaryotes. Here we show that a heretofore unrecognized pathway operates in yeast to regulate the transcription of *HAC1*. The resulting increase in Hac1p production, combined with the production or activation of a putative UPR modulatory factor, is necessary to qualitatively modify the cellular response in order to survive the inducing conditions. This parallel endoplasmic reticulum–to–nucleus signaling pathway thereby serves to modify the UPR-driven transcriptional program. The results suggest a surprising conservation among all eukaryotes of the ways by which the elements of the UPR signaling circuit are connected. We show that by adding an additional signaling element to the basic UPR circuit, a simple switch is transformed into a complex response.

## Abstract 2

The unfolded protein response in yeast was thought to be a simple on/off switch. Here, a second signaling element is revealed, transforming this simple switch into a complex response

## Introduction

In eukaryotes, the endoplasmic reticulum (ER) serves as the first station of the secretory pathway, through which all secreted and membrane proteins must pass. Within the ER, proteins are folded into their native structure and multisubunit protein complexes are assembled. The ER is a dynamic organelle, capable of sensing and adjusting its folding capacity in response to increased demand: when misfolded proteins accumulate in the ER, a signaling pathway, termed the unfolded protein response (UPR), is activated (reviewed in Ma and Hendershot 2001; Patil and Walter 2001; Kaufman 2002; Ron 2002). The UPR activates the expression of genes that enable the cell to adapt to and survive the stress, including those encoding ER-resident chaperones (Lee 1987; Kozutsumi et al. 1988), key enzymes in lipid biosynthesis (Cox et al. 1997), members of the ER-associated degradation (ERAD) machinery, and other components of the secretory system (Ng et al. 2000; Travers et al. 2000; Urano et al. 2000).

In yeast, the UPR is controlled by a binary switch imposed by a nonconventional splicing reaction that governs the production of the Hac1p transcription factor responsible for the activation of UPR target genes (Cox et al. 1993; Kohno et al. 1993; Cox and Walter 1996; Mori et al. 1992, 1996). In uninduced cells, direct base pairing between the 5′ untranslated region (UTR) and an intron at the 3′ end of the mRNA prevents *HAC1* mRNA translation (Chapman and Walter 1997; Ruegsegger et al. 2001). Accumulation of unfolded proteins activates the ER-resident transmembrane kinase/endoribonuclease Ire1p, which then cleaves the *HAC1* mRNA at two precise splice junctions, excising the intron (Cox et al. 1993; Mori et al. 1993; Sidrauski and Walter 1997). The two *HAC1* exons are then joined by tRNA ligase, allowing translation of Hac1p (Sidrauski et al. 1996).

To date, Ire1-dependent *HAC1* mRNA splicing is the only identified way by which signals from the ER lumen affect transcription in yeast. By contrast, in metazoan cells three mechanistically distinct pathways are known that operate in parallel, although their relative importance in different tissues remains to be determined (reviewed in Ma and Hendershot 2001). Hints that further complexity also exists in yeast comes from data presented in the accompanying paper (Patil et al. 2004): these data demonstrate that Hac1p activity is modulated by interaction with Gcn4p, a transcription factor central to regulation of amino acid biosynthesis. The UPR, therefore, may integrate signals from more than one source to compute a transcriptional output appropriate for the physiological conditions of the cell.

In this paper, we show that *HAC1* mRNA transcription is regulated, resulting in control of Hac1p abundance. Thus the on/off switch provided by *IRE1*-dependent splicing is not the only regulatory step of the UPR. This regulation responds to a bipartite signal that emanates from the ER and is communicated by an Ire1p-independent pathway. As a consequence, an alternate transcriptional program is triggered, with specific alterations to the normal UPR allowing the cell to survive. Thus, quantitative modulation of Hac1p imposes gain control on a binary switch in the UPR circuitry and, in collaboration with an additional signaling input, transforms a discrete transcriptional response into a more complex signaling function.

## Results

### Secretory Stress Boosts *HAC1* mRNA Abundance

**Figure 1:**
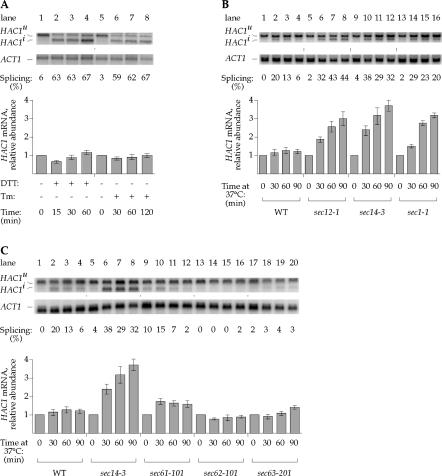
ER-Distal Secretory Stress Boosts *HAC1* mRNA Abundance (A) Determination of *HAC1* mRNA abundance during the UPR. The UPR was induced in WT cells by addition of either 6 mM DTT (lanes 1–4) or 1 μg/ml tunicamycin (lanes 5–8) for the times indicated. Total RNA was harvested at the indicated intervals, and the relative abundance of *HAC1* and *ACT1* mRNAs was analyzed by Northern blot analysis (see Materials and Methods). Splicing was calculated at the ratio of spliced *(HAC1^i^)* to total *(HAC1^i^ + HAC1^u^)* mRNA. (B) Determination of *HAC1* mRNA abundance during ER-distal secretory stress. WT, *sec12–1, sec14–3,* and *sec1–1* strains were grown at 23 °C and shifted to 37 °C. (C) Determination of *HAC1* mRNA abundance during ER-proximal secretory stress. WT, *sec14–3, sec61–101, sec62–101,* and *sec63–201* strains were grown at 23 °C and shifted to 37 °C.

To define the basic circuitry of signal transduction in the UPR, we evaluated the *HAC1* mRNA processing step in a quantitative manner. To this end, we induced the UPR with either dithiothreitol (DTT) or tunicamycin (both agents that cause protein misfolding selectively in the ER) and monitored *HAC1* mRNA by Northern blot analysis (Figure 1A). In agreement with previous results, we observed rapid and efficient splicing of *HAC1* mRNA, as apparent from the conversion of unspliced *HAC1^u^* mRNA (*u* for UPR-*u*ninduced) to spliced *HAC1^i^* mRNA (*i* for UPR-*i*nduced). Quantitation of the results shows that the relative abundance of *HAC1* mRNA (the sum of *HAC1^u^* and *HAC1^i^* mRNAs) remained unchanged over at least 12 h (Figure 1A; unpublished data). These data demonstrate that acute induction of unfolded proteins triggers a simple on/off switch that controls *HAC1* mRNA splicing.

In light of these observations, we were surprised to find that blocking the secretory pathway distal to the ER resulted in a pronounced increase in *HAC1* mRNA abundance. As shown in Figure 1B, *HAC1* mRNA levels increased 3- to 4-fold in mutant strains compromised at various steps in the secretory pathway when shifted to the nonpermissive temperature (*sec12–1:* ER → Golgi, lanes 5–8; *sec14–1:* intra-Golgi, lanes 9–12; and *sec1–1:* Golgi → plasma membrane, lanes 13–16) (Novick et al. 1980). Splicing was also induced, albeit to a lesser degree than was observed with DTT or tunicamycin treatment. The observed splicing suggests that blockages in ER-distal compartments of the secretory pathway lead to activation of Ire1p in the ER. Temperature shift alone only transiently induced *HAC1* mRNA splicing and had no effect on *HAC1* mRNA abundance (Figure 1B, lanes 1–4). To determine if any disruption of the secretory pathway had similar consequences, we blocked earlier stages of protein traffic. Mutations that blocked protein entry into the ER had no effect (Figure 1C: *sec62–101,* lanes 13–16; *sec63–201,* lanes 17–20) or only a mild effect (*sec61–101,* lanes 9–12) on *HAC1* mRNA abundance.

Thus, a surveillance pathway operates to adjust *HAC1* mRNA levels in response to altered conditions in the secretory pathway. In the experiments described above, we observed *HAC1* mRNA induction only in *sec* mutants that block transport distal to the ER, not in those that block protein entry into the ER. One common consequence of blocking the secretory pathway at later stages is that proteins in transit will eventually back up into the ER (Rose et al. 1989; Chang et al. 2002). This condition results in protein folding defects, thereby activating Ire1p, as indicated by the observed *HAC1* mRNA splicing. From the data discussed above (Figure 1A), however, we know that an accumulation of unfolded proteins alone is insufficient to trigger an upregulation of *HAC1* mRNA, suggesting that an additional inducing signal is required.

### *HAC1* mRNA Induction Requires a Bipartite Signal

**Figure 2:**
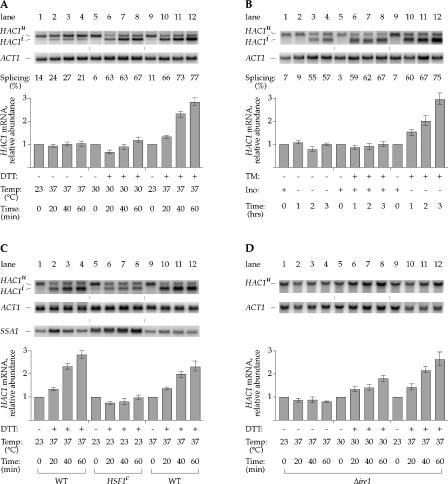
*HAC1* mRNA Induction Requires a Bipartite Signal and Is *IRE1*-Independent (A) Determination of *HAC1* mRNA abundance during ER stress and temperature shift. WT cells were grown at 23 °C and shifted to 37 °C (lanes 1–4 and 9–12) or kept constant at 30 °C (lanes 5–8). DTT was added as indicated (lanes 5–8 and 9–12). (B) Determination of *HAC1* mRNA abundance during ER stress and inositol deprivation. WT cells were grown at 30 °C in synthetic medium supplemented with inositol and shifted to synthetic medium lacking inositol (lanes 1–4 and 9–12), or continuously grown in medium supplemented with inositol (lanes 5–8). Tunicamycin was added to a final concentration of 1 μg/ml as indicated (lanes 5–8 and 9–12). (C) Distinction between heat shock response and *HAC1-*mRNA-inducing conditions. WT (lanes 1–4 and 9–12) and *HSF1^c^* (lanes 5–8) strains were grown at 23 °C and shifted to 37 °C (lanes 1–4 and 5–8) or continuously grown at 37 °C (lanes 9–12), and DTT added as indicated. (D) Analysis of *IRE1* pathway for a role in *HAC1* mRNA induction. Δ*ire1* cells were grown at 23 °C and shifted to 37 °C (lanes 1–4 and 9–12) or continuously grown at 30 °C (lanes 5–8), and DTT was added as indicated (lanes 5–8 and 9–12). Note that in Δ*ire1* cells, *HAC1* mRNA is modestly induced in response to DTT alone (lanes 5–8). This observation is indicative of feedback regulation, whereby a block in the UPR induces the I/T signal.

To determine the nature of this second signal, we sought conditions that induce *HAC1* mRNA when combined with ER protein misfolding drugs. Canvassing different conditions, we found two scenarios under which wild-type (WT) cells can be induced to upregulate *HAC1* mRNA: (1) ER protein misfolding combined with a temperature shift from 23 °C to 37 °C (Figure 2A) and (2) ER protein misfolding combined with inositol starvation (Figure 2B). Intriguingly, while ER protein misfolding and inositol starvation each activated the UPR individually (as shown by the activation of *HAC1* mRNA splicing; Figure 2A, lanes 5–8; Figure 2B, lanes 1–4 and 5–8), neither stress alone was sufficient to cause *HAC1* mRNA upregulation. Similarly, the temperature shift reproducibly caused a transient UPR induction (see Figure 1B, lanes 1–4; Figure 2A, lanes 1–4) but by itself did not affect *HAC1* mRNA levels. Only the combination of ER stress with either temperature shift (Figure 2A, lanes 9–12) or inositol starvation (Figure 2B, lanes 9–12) led to an increase in *HAC1* mRNA abundance. Subjecting cells to both temperature shift and inositol deprivation had no additive effect, nor did treating cells with both DTT and tunicamycin (unpublished data). Thus, *HAC1* mRNA induction requires a bipartite signal, consisting of one input provided by unfolded proteins in the ER (UP signal), and the other input provided by inositol starvation or temperature shift (I/T signal).

The heat shock response is transiently induced by shifting cells from 23 °C to 37 °C. To determine whether the heat shock response is an important component of the I/T signal, we tested whether continued growth at 37 °C or expression of a constitutively active allele of the heat shock factor Hsf1p (Sorger 1991; Bulman et al. 2001) would substitute for the temperature shift described above. Constitutive expression of active Hsf1p (Figure 2C, lanes 5–8) led to upregulation of *SSA1,* a known target of the heat shock response (Slater and Craig 1989), but did not substitute for the I/T signal for *HAC1* upregulation. In contrast, continued growth at 37 °C (Figure 2C, lanes 9–12) allowed for modest induction of *HAC1* mRNA. Thus, elevated temperature elicits effects other than heat shock, which are important for *HAC1* mRNA upregulation.

### *HAC1* Induction Is *IRE1*-Independent

The UP signal was experimentally induced by DTT or tunicamycin treatment of the cells. As Ire1p is a sensor of folding conditions within the ER lumen, we tested next whether Ire1p was required to transmit this signal. Surprisingly, it was not. *HAC1* mRNA abundance was induced 2.6-fold in Δ*ire1* cells (Figure 2D, lanes 9–12), similar to the 3-fold induction observed in WT cells (Figure 2A, lanes 9–12). These results show that a previously unrecognized Ire1p-independent surveillance mechanism must exist that monitors protein folding in the ER.

### *HAC1* mRNA Abundance Is Regulated Transcriptionally

**Figure 3:**
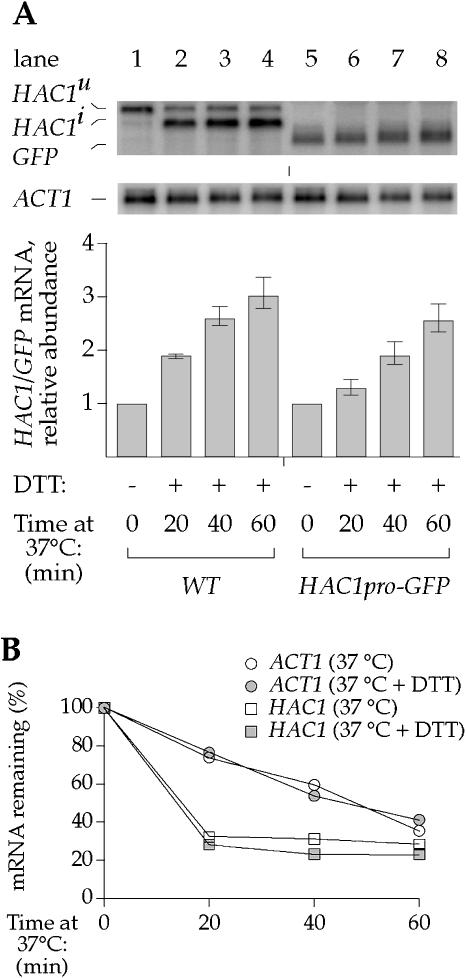
Activation of the *HAC1* Promoter Controls Increase in *HAC1* mRNA Abundance (A) Analysis of *HAC1* promoter activity during bipartite stress conditions. Δ*hac1* cells containing either a construct restoring *HAC1* expression (lanes 1–4) or a construct expressing *GFP* driven by the *HAC1* promoter (lanes 5–8) were grown at 23 °C and shifted to 37 °C concurrent with addition of DTT as indicated. (B) Determination of mRNA half-life during *HAC1*-mRNA-inducing conditions. *polII^ts^* cells were grown at 23 °C and were shifted to 37 °C either in the absence (open symbols) or presence (filled symbols) of DTT. *HAC1* mRNA abundance (squares) and *ACT1* mRNA abundance (circles) are normalized to the abundance of the PolIII transcript *SCR1*.

Increase of *HAC1* mRNA abundance could result from increased transcription, reduced degradation, or both. To distinguish between these possibilities, we constructed a reporter gene consisting of the *HAC1* promoter driving transcription of the open reading frame encoding the green fluorescent protein (GFP) flanked by *ACT1* untranslated regions *(HAC1pro-GFP).* The resulting heterologous *GFP* mRNA therefore contained no *HAC1* mRNA sequences. Under conditions providing both the UP and I/T signals, the change in abundance of the *GFP* mRNA (Figure 3A, lanes 5–8) mirrored that of the endogenous *HAC1* mRNA (Figure 3A, lanes 1–4), both in the kinetics and magnitude of the response. These data demonstrate that the observed increase in *HAC1* mRNA abundance was caused by increased transcriptional activity of the *HAC1* promoter.

To further test this notion, we compared the rate of decay of *HAC1* mRNA under both *HAC1*mRNA-inducing and noninducing conditions. To this end, we employed a strain bearing a temperature-sensitive allele of RNA polymerase II, which was subjected to either elevated temperature alone, or to both elevated temperature and DTT treatment. In both cases, polymerase II transcription ceased upon temperature shift, and mRNA decay was measured. As shown in Figure 3B, the rate of decay of *HAC1* mRNA was indistinguishable under the two conditions. Therefore, the increase in *HAC1* mRNA abundance in response to the combination of UP and I/T signals is due solely to activation of the *HAC1* promoter.

### *HAC1* Promoter Regulation Is Required to Survive Certain Stress Conditions

**Figure 4:**
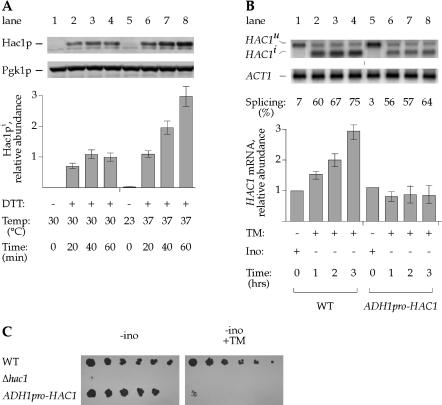
*HAC1* Promoter Regulation Is Required to Survive Stress (A) Determination of Hac1p levels during either ER stress alone or during both ER stress and temperature shift. WT cells were either grown at 30 °C and treated with DTT (lanes 1–4) or grown at 23 °C and simultaneously shifted to 37 °C and treated with DTT (lanes 5–8). Protein lysates were prepared, and protein levels were analyzed by Western blot analysis. The relative Hac1p/Pgk1p ratio is normalized to the DTT-treated sample (lane 4). (B) Characterization of *HAC1* expression in strain used to approximate basal *HAC1* expression. Cells expressing *HAC1* from the endogenous promoter (lanes 1–4) or the *ADH1* promoter (lanes 5–8) were grown at 30 °C in synthetic medium supplemented with inositol and shifted to synthetic medium lacking inositol simultaneous with the addition of tunicamycin. (C) Reduced viability of strains unable to express *HAC1* at elevated levels. The strains described in (B) were plated in serial dilutions (left to right) on synthetic medium lacking inositol (“−ino”) and synthetic medium lacking inositol and containing tunicamycin (“−ino +TM”).

The results presented so far define a novel regulatory mechanism whereby cells adjust the amount of *HAC1* mRNA. This mRNA is the substrate for the Ire1p-mediated splicing reaction, which in turn produces *HAC1^i^* mRNA that is translated to produce Hac1p transcription factor. We therefore asked whether elevated levels of *HAC1* mRNA led to a proportional increase in the level of Hac1p. Quantitative Western blot analysis showed that this is indeed the case: when cells were treated with DTT and concomitantly shifted to 37 °C, the levels of Hac1p increased 3-fold (Figure 4A, lanes 5–8), relative to the Hac1p levels observed in cells subjected to DTT treatment alone (Figure 4A, lanes 1–4). Therefore, the transcriptional induction of *HAC1* mRNA combined with Ire1p-mediated splicing results in elevated Hac1p levels, characterizing a new physiological state. Henceforth, we refer to this state as the “Super-UPR” (S-UPR).

To assess the physiological role of the S-UPR, we sought conditions that would allow us to directly monitor the consequences of changes in *HAC1* mRNA levels under otherwise identical growth conditions. To this end, we engineered a yeast strain unable to transcriptionally upregulate *HAC1*. In these cells, *HAC1* mRNA expression was removed from the control of the *HAC1* promoter and was instead driven by the heterologous *ADH1* promoter (*ADH1pro*-*HAC1*), at levels closely approximating the uninduced *HAC1* state (Figure 4B, compare *ADH1pro*-*HAC1*, lanes 5–8, to *HAC1pro*-*HAC1*, lanes 1–4). Expression from the *ADH1* promoter was constitutive, and the levels of *HAC1* mRNA did not change significantly under the various inducing conditions described above. As expected, induction of the UPR in these strains led to efficient *HAC1* mRNA splicing and Hac1p production. This strain therefore allowed us to fix the cellular Hac1p concentration to a level closely approximating the basal *HAC1* expression state observed during the UPR.

We next assessed whether we could identify physiological conditions under which elevated *HAC1* mRNA levels were required for cell growth. Therefore, we subjected WT cells and the engineered strain described above to the combinations of stresses described in Figure 2. Cells expressing *HAC1* from the endogenous or from the *ADH1* promoter grew equally well on plates lacking inositol (Figure 4C, left, first and third rows). This condition induces the UPR and requires the expression of at least a minimal amount of *HAC1* mRNA, as Δ*hac1* cells fail to grow (Figure 4C, left, second row). In contrast, only WT cells, which are able to upregulate *HAC1* mRNA production, grew on plates lacking inositol and also containing tunicamycin. Cells expressing *HAC1* mRNA only at the basal levels from the *ADH1* promoter were nonviable on these plates (Figure 4C, right, third row). As shown previously in Figure 2B, this combination of stresses induces the S-UPR. The data therefore reveal that regulation provided by the *HAC1* promoter is necessary for cells to survive certain stress conditions that otherwise are lethal.

### Differential UPR Target Gene Induction by Elevated Hac1p Levels

To begin to characterize the cause for increased viability, we next determined differences in UPR target gene expression resulting from either UPR or S-UPR induction.

**Figure 5:**
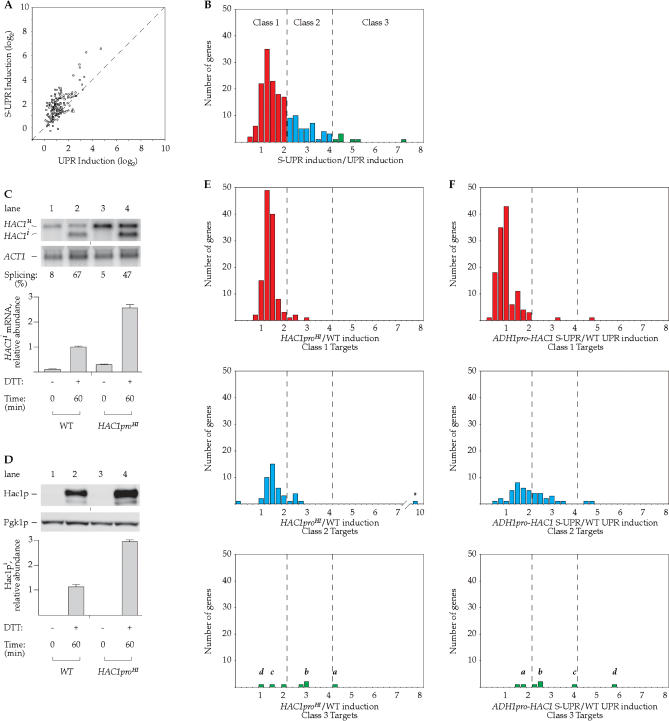
Differential UPR Target Gene Induction by Elevated Hac1p Levels (A) Comparison of UPR target gene induction under either UPR or S-UPR conditions. Whole-genome mRNA expression analysis was carried out on WT cells harvested after 60 min of treatment, either grown at 30 °C and treated with 6 mM DTT (x-axis), or grown at 23 °C and simultaneously shifted to 37 °C and treated with 6 mM DTT (y-axis). Fold changes in gene expression are in reference to the untreated (*t* = 0) samples. Shown are only those genes designated as targets of the UPR (see Materials and Methods). The dashed diagonal line represents equal induction under both conditions. (B) Comparison of UPR target gene induction under either UPR or S-UPR conditions (alternate display). The data from (A) were analyzed to generate a ratio (x-axis) for each gene, dividing the induction during S-UPR-inducing conditions by the induction during UPR-inducing conditions, with target genes of similar ratio grouped together (y-axis). (C) Characterization of *HAC1* expression in a strain constitutively expressing *HAC1* at high levels. Cells expressing *HAC1* from the endogenous promoter (WT; lanes 1 and 2), or a modified promoter constitutively expressing *HAC1* at high levels (*HAC1pro^HI^*; lanes 3 and 4) were treated with 6 mM DTT for 60 min. Although the basal transcription of *HAC1pro^HI^* is elevated, the promoter is still capable of further induction during the S-UPR (unpublished data). (D) Determination of Hac1p level in a strain constitutively expressing *HAC1* at high levels. Protein lysates were prepared from the strains described in (C), and protein levels were analyzed by Western blot analysis. The relative Hac1p/Pgk1p ratio is normalized to the WT DTT-treated (*t* = 60) sample from Figure 4A. (E) Transcriptional response of different classes of UPR targets to high levels of Hac1p. Whole-genome mRNA expression analysis was carried on *HAC1pro^HI^* and WT cells treated with 6 mM DTT and harvested after 60 min. For the genes in each of the three classes of UPR targets defined in (B), a ratio (x-axis) is calculated by dividing the fold induction in DTT-treated *HAC1pro^HI^* cells by the fold induction in DTT-treated WT cells. This ratio is plotted against the number of genes with a similar ratio (y-axis). The Class 2 target *YFR026C* (asterisk), which is DTT-induced approximately 10-fold more in *HAC1pro^HI^* than in WT cells, is of unknown function. *a, DER1; b, INO1; c, YOR289W; d, YHR087W*. (F) Transcriptional response of different classes of UPR targets to UMF. Whole-genome mRNA expression analysis was carried on *ADH1pro-HAC1* cells grown at 23 °C and simultaneously shifted to 37 °C and treated with 6 mM DTT, and WT cells treated with 6 mM DTT, both harvested after 60 min. For the genes in each of the three classes of UPR targets defined in (B), a ratio (x-axis) is calculated by dividing the fold induction in *ADH1pro-HAC1* cells under S-UPR-inducing conditions by the fold induction in WT cells under UPR-inducing conditions. This ratio is plotted against the number of genes with a similar ratio (y-axis). *a, DER1; b, INO1; c, YOR289W; d, YHR087W*.

To this end, we used DNA microarray chip analysis to determine the complete mRNA profile of cells grown under UPR and S-UPR conditions. The results of this analysis are shown in Figure 5A. Each spot represents the fold induction of a UPR target under UPR conditions (x-axis) or S-UPR conditions (y-axis) (see Materials and Methods for definition of the UPR target set used in this analysis). UPR target genes for which the S-UPR has no additional effect should undergo equal induction under both conditions, and are expected to scatter around the diagonal, indicated by the dashed line. This was the case for many UPR targets. However, induction of a substantial number of genes was skewed to the top of the graph, indicating stronger induction under S-UPR conditions than under UPR conditions. These same data are displayed in Figure 5B to highlight and categorize these differences. In the histogram, the x-axis represents the ratio of the induction of a target gene during S-UPR and UPR conditions, and the y-axis shows the number of genes with a given ratio. We have operationally divided UPR target genes into three classes, based on their fold induction during the S-UPR compared to their fold induction during the UPR. (1) Class 1 targets (Figure 5, red bars) exhibit little if any difference in induction during the UPR and S-UPR (S-UPR induction / UPR induction < 2). Thus, the increased Hac1p during the S-UPR does not lead to enhanced transcription, indicating that for these genes the response is already saturated at UPR Hac1p levels. Class 1 targets include many of the known genes encoding ER lumenal chaperones (including *KAR2, SCJ1, LHS1,* and *JEM1*) and redox proteins (including *PDI1, EUG1,* and *ERO1*). (2) Class 2 targets (Figure 5, blue bars) are induced to a 2- to 4-fold greater extent during S-UPR than during the UPR. Transcription of these genes is therefore roughly proportional to the Hac1p levels in the cell. Class 2 targets include *YIP3,* involved in ER-to-Golgi transport, *OPI3,* encoding a phospholipid methyltransferase, and the hexose transporters *HXT12, HXT15, HXT16,* and *HXT17*. (3) Class 3 targets (Figure 5, green bars) are induced by the S-UPR greater than 4-fold more than by the UPR. Class 3 contains the UPR targets *DER1,* involved in ER-associated degradation (Knop et al. 1996; Ng et al. 2000; Travers et al. 2000), and *INO1,* critical for membrane biogenesis (Hirsch and Henry 1986).

### Role for a Putative UPR Modulatory Factor

The increased transcriptional output under S-UPR conditions could occur for two reasons. It could be due to increased Hac1p concentrations in the cell, or it could result because an additional S-UPR-specific transcription factor is produced or activated (perhaps the same that regulates *HAC1* transcription). It could also be due to a combination of these two scenarios. To distinguish among these possibilities, we determined the target gene induction profile in cells in which the *HAC1* mRNA concentration was artificially elevated to a similar level as that found after S-UPR induction. We took advantage of a specific 15-bp deletion in the *HAC1* promoter *(HAC1pro^HI^),* which increases basal expression by about 3-fold, as compared to the endogenous promoter (Figure 5C). In cells bearing a *HAC1pro^HI^*-*HAC1* gene (“*HAC1pro^HI^* cells”), splicing of *HAC1* mRNA was somewhat reduced upon UPR induction (47%, compared to 67% for WT); however, even with this reduction, *HAC1pro^HI^* cells produced approximately 2.5-fold more spliced *HAC1^i^* mRNA than WT cells (Figure 5C, compare lanes 3 and 4 to lanes 1 and 2). The increased levels of *HAC1^i^* mRNA led to a corresponding increase in Hac1p (Figure 5D, compare lanes 3 and 4 to lanes 1 and 2). The amount of Hac1p produced by DTT induction of *HAC1pro^HI^* cells is approximately the same as the amount of Hac1p produced during the S-UPR (compare Figure 5D, lanes 2 and 4 with Figure 4A, lanes 4 and 8).

The ability to set *HAC1* mRNA levels to S-UPR levels allowed us to compare directly UPR target gene induction with the cellular Hac1p concentration being the only variable. We induced the UPR in both WT and *HAC1pro^HI^* cells with DTT and determined the mRNA expression profiles. For each class of UPR target defined above, the expression analysis of UPR-induced WT and *HAC1pro^HI^* cells is shown in Figure 5E. In the histograms, the x-axis shows the ratio of target gene induction during the UPR driven by a high level of Hac1p from *HAC1pro^HI^* cells compared to induction during the UPR in WT cells. The y-axis shows the number of genes at any given ratio. As expected, Class 1 targets (Figure 5E, top panel) did not further respond to the higher levels of Hac1p produced in *HAC1pro^HI^* cells. The majority of Class 2 and Class 3 targets (Figure 5E, middle and bottom panels) also did not respond to higher levels of Hac1p (ratio less than 2), indicating that only raising the Hac1p concentration in cells is not sufficient to account for their full increased induction during the S-UPR. By contrast, ten of the 32 Class 2 and Class 3 targets were significantly induced (ratio greater than 2) in cells expressing high levels of Hac1p. For the Class 3 target *DER1,* high levels of Hac1p were sufficient to elevate expression to S-UPR levels (compare 8-fold induction in DTT-treated *HAC1pro^HI^* cells to 9-fold induction in WT cells during the S-UPR). Otherwise, however, high levels of Hac1p did not fully reconstitute the induction seen during the S-UPR. For example, while the Class 3 gene *INO1* was induced 7.5-fold more in the S-UPR than in the UPR, it was induced only 3-fold more by high levels of Hac1p, compared to normal levels. We conclude that elevated Hac1p levels are sufficient to selectively increase the induction of a few UPR targets, but that the full transcriptional program of the S-UPR predicts the production or activation of an additional transcriptional activator, which we term UPR modulatory factor (UMF).

To dissect further the UMF contribution during the S-UPR, we sought conditions under which UMF activity was the only variable. To this end, we induced the S-UPR in *ADH1pro*-*HAC1* cells, which are prevented from achieving high level Hac1p expression, and compared the mRNA expression profile against the UPR in WT cells. In this analysis, Hac1p levels were approximately equivalent in the two conditions, so variations from the normal UPR transcriptional program reflect the activity of UMF. The results are shown in Figure 5F, with the data displayed similarly to Figure 5E: the x-axis shows the ratio of target gene induction during the S-UPR in *ADH1pro*-*HAC1* cells, compared to induction during the UPR in WT cells, and the y-axis shows the number of genes at any given ratio. Not surprisingly, the induction of Class 1 targets (Figure 5F, top panel) was unaffected: these are targets that are fully induced by even low levels of Hac1p and are not more induced during the S-UPR. Two Class 3 targets, *YOR289W* and *YHR087W* (both of unknown function) reach near WT S-UPR induction levels, without elevated levels of Hac1p; for these targets, UMF likely plays a leading role in their induction, with Hac1p having less influence. Most Class 2 and Class 3 targets (Figure 5F, middle and bottom panels), however, do not reach full S-UPR induction levels in the absence of elevated Hac1p levels. For example, the Class 3 target *INO1* is induced roughly 25-fold in *ADH1pro*-*HAC1* cells during S-UPR conditions; while this is roughly twice the induction observed during the UPR, it falls far short of the 75-fold S-UPR induction in WT cells.

These results reinforce the in vivo requirement for high levels of Hac1p to survive S-UPR stress, demonstrated in Figure 4C. Taken together with the data shown in Figure 5E, we conclude that the full S-UPR transcriptional program results from a collaboration between elevated Hac1p levels and UMF, with the relative contribution from each varying among different target genes.

## Discussion

### The Circuitry of the UPR

In this paper, we describe a novel ER surveillance pathway in yeast that modulates the UPR, resulting in a new physiological state that we term the S-UPR. In response to a bipartite signal transmitted from the ER by an *IRE1*-independent pathway, the *HAC1* promoter is activated, resulting in increased *HAC1* mRNA levels that, upon splicing, yield more Hac1p. The increased Hac1p concentration, in conjunction with an additional postulated factor(s) produced or activated by the S-UPR (UMF), allows the cell to mount a modified transcriptional response to cope with the inducing stress conditions.

**Figure 6:**
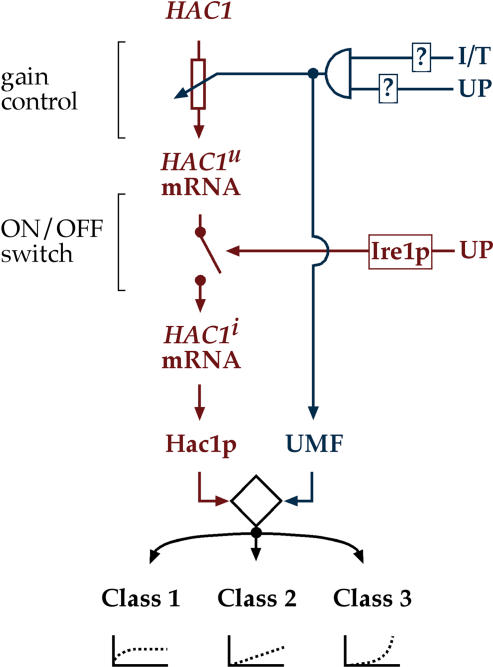
A Schematic of the Circuitry of the UPR The model depicts the circuitry of the UPR (red) and the S-UPR (blue). Transcriptional control of *HAC1* is indicated by an icon representing a rheostat affording gain control of the UPR; Ire1p-dependent *HAC1^u^* mRNA splicing is indicated by an icon representing an on/off switch. The I/T and UP signals in the S-UPR are integrated by an AND gate (semicircle, top right), i.e., both conditions must be met to propagate the S-UPR signal. The putative UMF may collaborate with Hac1p to control transcription of UPR target genes (shown) and also be involved in regulating *HAC1* transcription (not shown); alternatively, different factors may be involved. The collaboration of Hac1p and UMF is indicated by the diamond-shaped icon, which integrates the information coming from both Hac1p and UMF concentration and activity.

Figure 6 shows the UPR as a circuit diagram utilizing multiple logical operations to integrate various signals. In the “classical UPR” (in red), basal transcription of *HAC1* produces *HAC1^u^* mRNA, which is translationally inactive due to the presence of the inhibitory intron. In response to unfolded proteins, Ire1p performs an on/off operation, excising the intron from *HAC1^u^* mRNA to generate spliced *HAC1^i^* mRNA, which is translated to produce the Hac1p transcription activator. The S-UPR provides another layer of regulation superimposed on the UPR (in blue). If ER folding stress is combined with either a shift to elevated temperature or inositol starvation, an AND gate integrates this bipartite signal and boosts *HAC1* mRNA levels. In turn, this regulation causes increased Hac1p production. Together with UMF, Hac1p induces UPR target genes, with particular genes responding differentially to differences in Hac1p and UMF concentration. Thus the S-UPR can be seen as an adaptation of the classical (or basal) UPR, fine-tuning the activation of select targets to produce a response suited to the challenge faced by the cell.

In the accompanying paper, Patil et al. (2004) describe a third signaling element, which additionally modifies the transcriptional program of the yeast UPR. The authors show that the transcriptional activator Gcn4p collaborates with Hac1p at the promoters of UPR targets, providing an additional opportunity for integration of information about the physiological state of the cell. Gcn4p is a highly regulated transcription regulator that responds to metabolic conditions, such as amino acid availability. Gcn4p is not UMF, as S-UPR induction of *HAC1* proceeds normally in Δ*gcn4* cells (unpublished data). A recent report from Ogawa and coworkers (Ogawa and Mori 2004) demonstrates autoregulation of *HAC1* expression under conditions of extreme and prolonged ER stress, mediated by Hac1p binding to its own promoter. Because the S-UPR can be triggered in Δ*ire1* cells that do not produce Hac1p, autoregulation and the S-UPR are distinct pathways. The existence of multiple mechanisms of *HAC1* regulation reinforces the notion that multiple cellular stimuli become integrated to fine-tune an appropriate response.

### Bipartite Signal Requirement for S-UPR Activation

Presently, the molecular details of the pathway by which the S-UPR signal exerts transcriptional control are not known. In particular, it will be of interest to determine where in the cell the two branches of the S-UPR signal are integrated, i.e., how the AND gate is constructed and where it resides. One possibility is that this signal integration event occurs close to the source at the ER membrane. Both temperature shift and inositol starvation can equally induce the I/T signal pathway, and it is conceivable that both conditions affect ER membrane properties similarly. Inositol is an essential precursor for phosphatidylinositol, a major structural phospholipid in yeast that is required for proper functioning of the secretory system (White et al. 1991; Zinser and Daum 1995; Greenberg and Lopes 1996). Previous work has demonstrated an intimate link between inositol regulation and the UPR, presumably to coordinate the concentration of ER lumenal and membrane components (Cox et al. 1997). A similar sensing mechanism operates in cholesterol homeostasis, with sterol composition in ER membranes affecting the activity of SCAP, a membrane-bound regulator of SREBP intramembrane proteolysis (Espenshade et al. 2002). It is likely that elevated temperatures also affect ER membrane properties, such as fluidity (Laroche et al. 2001). If such a property were sensed, it would explain how the temperature effect contributing to the I/T signal is separate from the heat shock response. ER membranes distressed by either inositol deprivation or elevated temperature (the I/T signal) might then control the activity of a membrane-bound component of a signal transduction machine that also senses protein folding conditions (the UP signal) in the ER lumen.

Alternatively, the AND gate might be well removed from the ER membrane, with I/T and UP signals traveling separately through the cell and meeting possibly as late as at the promoters of the affected target genes. Components that map onto either signaling pathway need to be identified and placed into the circuit to distinguish between these possibilities.

### The Transcriptional Output of the S-UPR

The transcriptional response elicited by the S-UPR reveals different classes of UPR targets. During the S-UPR, the further activation of UPR targets is not simply proportional to the increase in Hac1p concentration; rather, we observe a multitude of complex responses. Some targets are already maximally transcribed during UPR conditions and are not induced further during the S-UPR, while other targets become significantly more induced. For some targets (a minority), elevated Hac1p concentrations are sufficient to increase transcriptional induction, while for others, S-UPR-derived UMF is also required. We find evidence for both kinds of regulation. The promoters of target genes, therefore, display differential responsiveness to Hac1p concentration and UMF activity.

The production of different levels of Hac1p allowed us to isolate and directly assess the responsiveness of target genes to Hac1p concentration under otherwise identical conditions. Those target genes that undergo equivalent activation under both conditions likely have promoters that are saturated by the lower amount of Hac1p, and thus reach full activation more readily. For UPR targets at the other end of the spectrum, induction continues to increase as Hac1p levels increase; lower concentrations of Hac1p are inadequate for full stimulation of these genes, which may have lower affinity for Hac1p. Because genes respond differentially to Hac1p levels, regulation of *HAC1* mRNA abundance can be used as a gene-specific gain control for target activation. This control is similar to that observed in regulation of phosphate metabolism, where the differential affinity of certain Pho4p phosphoforms for target promoters allows for the selective activation of a subset of phosphate-responsive genes (Springer et al. 2003).

For most target genes, however, the S-UPR further enhances the transcriptional activity even in the presence of high concentrations of Hac1p. For example, *INO1* is induced over 75-fold by the S-UPR in WT cells, compared to 33-fold during the UPR in *HAC1pro^HI^* cells, while the amount of Hac1p produced in both cases is approximately the same. This added induction during the S-UPR is dependent on Hac1p, as *ADH1pro*-*HAC1* cells treated with DTT and shifted to elevated temperature show significantly reduced induction of *INO1*. The simplest interpretation of these findings is that S-UPR-induced UMF, which may or may not be identical to the transcription factor regulating *HAC1* mRNA, collaborates with Hac1p to further boost transcription of these genes.

The *cis* determinants that instruct genes to behave as Class 1, 2, or 3 targets are unknown. One attractive possibility is that target gene promoters have differential affinity for Hac1p and/or UMF. Promoters with stronger affinity for Hac1p would be maximally occupied and fully activated during a normal UPR and would not further respond to increased Hac1p levels (i.e., Class 1 targets). Promoters with lesser affinity for Hac1p would increase in occupancy, and hence transcriptional activation, as Hac1p levels rose during the S-UPR, and would possibly achieve full transcriptional activity only with the additional binding of UMF (i.e., Class 2 and 3 targets). Such a mechanism of promoter-encoded differential responsiveness to transcription factor concentration would explain the selective regulation of subsets of UPR target genes.

### Links with the Metazoan UPR

Higher eukaryotes possess three separate pathways to sense ER stress and direct overlapping but distinct transcriptional outputs (reviewed in Ma and Hendershot 2001). In the first branch, Ire1p senses unfolded proteins in the ER lumen and directs the cleavage of an intron from the mRNA encoding the XBP-1 transcription factor, analogous to the splicing of *HAC1* in yeast (Yoshida et al. 2001; Calfon et al. 2002). In a second branch, the transmembrane kinase PERK phosphorylates and inactivates the eIF2-α translation initiation factor (Harding et al. 1999). This attenuates global protein synthesis, but selectively increases the translation of a small number of proteins including the ATF-4 transcriptional activator. Interestingly, ATF-4 is the metazoan ortholog of Gcn4p, the yeast transcription factor demonstrated by Patil et al. (2004) to collaborate with Hac1p. Finally, in a third branch, activation of the UPR in metazoans allows for the ER-to-Golgi transit of the membrane-tethered ATF-6 protein. In the Golgi apparatus, ATF-6 undergoes proteolytic cleavage within its membrane-spanning domain, and the soluble fragment subsequently travels to the nucleus as an active transcription factor (Haze et al. 1999; Ye et al. 2000). XBP-1, ATF-4, and ATF-6 all activate separate but overlapping transcriptional programs that enable the cell to respond to changing conditions in the ER. Notably, the XBP-1 promoter is a target of ATF-6 activation (Yoshida et al. 2001), reminiscent of the circuitry described here for yeast. Conceptually, therefore, *HAC1* mRNA upregulation by the S-UPR pathway in yeast takes the place of XBP-1 upregulation by the ATF-6 fragment in metazoans. Moreover, ATF-6 and XBP-1 can heterodimerize (Lee et al. 2002), reminiscent of the proposed collaboration of UMF and Hac1p. Thus, intriguing parallels between yeast and metazoans in the wiring that connects the elements of the UPR signaling circuit are beginning to come to light.

These findings suggest a common strategy among all eukaryotic cells for responding to challenges to the secretory system. Maintaining separate ER surveillance pathways creates the potential for cells to integrate multiple signals that, in principle, could convey precise information regarding the nature of the imbalance to afford finely tailored corrective measures. In this view, the UPR operates as a homeostatic control circuit, in which such regulation ensures that components of the secretory apparatus are produced according to need. The challenge now at hand is to decipher the logic between the UPR inducing conditions and the transcriptional output to add physiological explanations to the complex regulation of the response that we observe experimentally.

## Materials and Methods

### 

#### Yeast strains.

The WT strain W303–1A, the Δ*ire1* strain CS165, and the Δ*hac1* strain JC408 are as described previously (Cox et al. 1993; Cox and Walter 1996). All *sec* strains used in this study were provided by Robert Fuller (University of Michigan, Ann Arbor, Michigan, United States) and are otherwise genotypically identical to W303. The *HSF1^c^* strain was a kind gift of Hillary Nelson (University of Pennsylvania, Philadelphia, Pennsylvania, United States) and contains the R222A allele of *HSF1* (Bulman et al. 2001) replacing the chromosomal locus in a W303 background. Strains used in the experiments described in Figure 3A were Δ*hac1* transformed with pPW598 (*HAC1pro-HAC*1*, HA*-tagged *HAC1* [Cox and Walter 1996] under its own promoter and with native *HAC1* flanking sequences, in a pRS304 background) or with pPW599 *(HAC1pro-GFP,* the *GFP* ORF, driven by the *HAC1* promoter [defined as the region starting at the mapped start site of *HAC1* transcription (Ruegsegger et al. 2001) and extending 500 bp upstream] and flanked by 5′ UTR and 3′ UTR sequences from *ACT1).* Strains used in experiments described in Figure 4 were *HAC1pro-HAC1* and Δ*hac1* (described above) and Δ*hac1* transformed with pPW600 (*ADH1pro-HAC1, HA*-tagged *HAC1* with 5′ and 3′ UTR *HAC1* sequence subcloned into the p414 ADH expression vector [Mumberg et al. 1995] and transferred to a pRS304 backbone). In Figure 5, *HAC1pro^HI^* (pPW601) was made by subjecting *HAC1pro-HAC1* to QuikChange mutagenesis (Stratagene, La Jolla, California, United States) following the manufacturer's protocol, using oligonucleotides to remove the 15 bp at coordinates −338 to −323 (+1 representing the start site of transcription).

#### Cell culture and plates

Yeast cultures were grown in YPD medium (unless otherwise specified) at the indicated temperatures to midlog phase (OD_600_ **≈** 0.5). For temperature shift experiments, cultures were transferred to a preheated 37 °C water bath shaking incubator. DTT (Roche, Basel, Switzerland) was added to a final concentration of 6 mM, and tunicamycin (Boehringer Mannheim, Indianapolis, Indiana, United States) was added to a final concentration of 1 μg/ml. For experiments involving inositol deprivation in liquid medium, yeast cells were grown in liquid complete synthetic medium described by Sherman (1991), supplemented with myo-inositol (Sigma, St. Louis, Missouri, United States) to a final concentration of 100 μg/ml. Cells were then harvested by filtration, washed three times in prewarmed complete synthetic medium lacking inositol, and then filter-transferred to a flask containing prewarmed complete synthetic medium lacking inositol.

For the experiment described in Figure 4C, yeast strains were grown in YPD to midlog phase (OD_600_ ≈ 0.5), transferred to a 96-well microtiter plate, and serially 5-fold diluted in fresh YPD. Using a liquid transfer prong (“frogging”) tool (Aladin Enterprises, San Francisco, California, United States), approximately 3 μl of all serial dilutions of all strains was simultaneously transferred to complete synthetic plates lacking inositol (described above), either in the absence or presence of 0.2 μg/ml tunicamycin. After approximately 2 d of incubation at 30 °C, plates were photographed using the Epi Chemi II Darkroom GelDoc system (UVP, Upland, California, United States).

#### RNA analysis

Isolation of total RNA from yeast cells was carried out with the modified hot-phenol extraction method described in Ruegsegger et al.(2001). For Northern blot analysis, 10 μg of total RNA was separated on a 1.5% w/v agarose gel and transferred to a Duralon-UV nylon membrane (Stratagene), which was incubated with a probe directed against the 5′ exon of *HAC1*. The mRNA abundance was quantitated using a PhosporImager (Molecular Dynamics, Sunnyvale, California, United States). The membranes were then stripped with two serial washes using 0.1% SDS at 65 °C for 60 min each and incubated with a probe directed against the 3′ exon of *ACT1,* and mRNA abundance was again quantitated. To control for the variable strength of Northern blot probes across multiple experiments, the relative *HAC1*/*ACT1* mRNA abundance ratio is always normalized to the untreated (*t* = 0) sample. For the detection of other mRNAs, membranes were incubated with the additional relevant probes *(GFP, SSA1)* concurrent with the *HAC1* probe. All data shown are an average of at least two independent experiments.

PolyA+ mRNA was isolated from total RNA using the PolyATract system (Promega, Madison, Wisconsin, United States) according to the manufacturer's instructions. Microarray analysis, using yeast ORF arrays printed at the University of California, San Francisco, Core Center for Genomics and Proteomics (http://derisilab.ucsf.edu/core/, was performed as in Carroll et al. (2001) using protocols and reagents described at http://microarrays.org/. All array data are the average of two independent experiments. For this study, we were obliged to evaluate UPR targets differently than in Travers et al. (2000), as we considered *HAC1*-independent responses, whereas the former study specifically isolated genes induced by unfolded proteins via Hac1p (*z-*score ≥ 3.6 σ). Here, UPR targets were defined as those genes that met the following three criteria in a parallel set of microarray experiments using WT (W303) and Δ*hac1* (JC408) strains. First, induction (log_2_ of the fold change in gene expression) in WT cells treated with DTT must be at least one standard deviation greater than the mean ([*induction* ^WT,DTT^ − *μ* ^WT,DTT^]/*σ* ^WT,DTT^ ≥ 1). Second, induction in WT cells treated with tunicamycin must be at least one standard deviation greater than the mean ([*induction* ^WT,tunicamycin^ − *μ* ^WT,tunicamycin^]/*σ* ^WT,tunicamycin^ ≥ 1). Third, induction in Δ*hac1* cells treated with DTT must be at least one standard deviation less than the induction in WT cells treated with DTT (or, more awkwardly, [([(*induction* ^WT,DTT^ − *μ* ^WT,DTT^)/*σ* ^WT,DTT^] − [(*induction* ^Δ*hac1*^ ^,DTT^ − *μ* ^Δ*hac1*^ ^,DTT^)/*σ* ^Δ*hac1*,DTT^]) − *μ* ^WT,DTT − Δ*hac1*,DTT^]/*σ* ^WT,DTT − Δ*hac1*,DTT^ ≥ 1).

#### Isolation and detection of protein from yeast cells

Cells were collected by filtration, frozen in liquid nitrogen, and disrupted in 150 μl of 8 M urea/1% SDS by vortexing for 5 min at 4 °C in the presence of 150 μl of silica beads. The samples were then boiled for 5 min and the lysates cleared by centrifugation at 16,200*g* for 5 min at room temperature. SDS-PAGE was performed on 20 μg of protein separated on NuPAGE 10% w/v SDS-polyacrylamide gels (Invitrogen, Carlsbad, California, United States), and Western blots were visualized using SuperSignal West Dura Extended Duration ECL Substrate (Pierce Biotechnology, Rockford, Illinois, United States) according to the instructions of the manufacturer. Hac1p was detected using a polyclonal antibody raised against the carboxy terminus (see Figure 4) or a monoclonal antibody raised against the HA epitope and directly coupled to horseradish peroxidase (see Figure 5) (Molecular Probes, Eugene, Oregon, United States), and Pgk1p was detected using a commercially available polyclonal antibody (Molecular Probes). Protein abundance was quantified using the Epi Chemi II Darkroom GelDoc system (UVP). Parallel experiments using serial protein dilutions were performed to confirm that the detected protein levels were within the linear range of the system.

#### Transcription shut-off

The yeast strain JC218 (Sidrauski et al. 1996; *rbp1–1*) was grown in YPD at 23 °C to OD_600_ ≈ 0.5 and then shifted to a 37 °C water bath, shaking at 250 RPM. To induce the UPR, DTT was added to a final concentration of 6 mM. Cells were harvested and total RNA isolated, at 20 min intervals, as described above.

## Supporting Information

### Accession Numbers

The GenBank accession numbers of the sequences discussed in this paper are Hac1p (NP_ 116622), Ire1p (NP_011946), and tRNA ligase (NP_012448).

Microarray data can be accessed at the Gene Expression Omnibus (GEO) at the National Center for Biotechnology Information (NCBI) database as platform number GPL999 and sample numbers GSM16978–GSM1984.

## Abbreviations

DTT: dithiothreitol
ER: endoplasmic reticulum
GFP: green fluorescent protein
I/T signal: signal provided by inositol starvation or temperature shift
S-UPR: Super-UPR
UMF: unfolded protein response modulatory factor
UP signal: signal provided by unfolded proteins in the ER
UPR: unfolded protein response
UTR: untranslated region
WT: wild-type
